# Personals

**Published:** 1913-12

**Authors:** 


### PERSONALS

*Announcement of removal, travel, and other matters of interest are requested. Please report errors in the listing of any physician in the State and other directories that we may co-operate with the State Society in securing a correct list.*

Dr. Carl A. Blackley has been appointed medical inspector in the schools of Lockport. The Board of Education have formally demanded of the Common Council the restoration of an item of $1200 salary, in accordance with the new State law, but vetoed by the Mayor.

Dr. Joseph C. O'Gorman of Buffalo, president of the New York State Civil Service Association, was tendered a reception at a smoker given by the Buffalo branch, October 31.

Dr. Horace Lo Grasso, Supt. of the J. N. Adam Memorial Hospital at Perrysburg, described the work of the hospital for incipient tuberculous patients at the November meeting of the Nurses' Association of Buffalo.

Dr. Harry A. Wood has removed from Buffalo to Glenwood, N. Y.

Dr. Lesser Kauffmann and Herbert M. Hill, Ph. D., of Buffalo, spent a couple of weeks hunting in the Adirondacks in November.

Dr. A. G. Pohlman, a former Buffalo boy, son of the late Prof. Julius Pohlman, has been elected head of the Dept. of Anatomy in St. Louis University.

Dr. Prescott Le Breton of Buffalo announces the removal of his office to 125 Allen street.

Dr. Max Breuer of Buffalo gave a dinner at his home on October 25, in honor of Prof. Adolph Schmidt of Halle and Dr. Goetz of Blamkenburg, Thüringen.

Dr. Frank W. Love of Buffalo has moved to 470 Linwood avenue.

Dr. George H. Westinghouse of Buffalo has recently purchased and occupied, as residence and office, the commodious house situated at 2830 Main street.

Dr. S. S. McKenzie has recently moved from Bolivar, N. Y., to 413 E. Market St., Warren, Ohio.

Dr. Henry C. Buswell of Buffalo has returned from Europe.

Major Wm. G. Bissell, M. D., of Buffalo, retiring after 25 years' service from the 74th Reg., was honored by a review of the regiment, November 25.

Dr. J. Henry Dowd of Buffalo will read a paper, by special invitation, before the New York Academy of Medicine, at the January meeting. His subject is "The Phosphatic Index, Its Relation to Diagnosis and Treatment."

Dr. Sanford H. Kinney of New York, formerly of the staff of the State Soldiers' Home at Bath, recently visited Bath.

Dr. L. G. Visscher of Los Angeles, who is making a tour of this country, spent a couple of days in Buffalo late in November. He is especially interested in gastro-enterology.

Dr. F. W. Zimmer was elected a school commissioner of Rochester.

Dr. Thos. A. Killip was re-elected a coroner of Monroe County.

---

Dr. G. W. Goler, Dr. Joseph Roby and Dr. Marion Craig Potter of Rochester read papers before the State Conference of Sanitary Officers at Utica, November 19 to 21.

---

Dr. Hugh Cabot of Boston was in Rochester November 14 and 15 and spoke on "Sex Hygiene"—when, where and how shall sex instruction be given?

